# HYPER RECOMBINATION1 of the THO/TREX Complex Plays a Role in Controlling Transcription of the *REVERSION-TO-ETHYLENE SENSITIVITY1* Gene in Arabidopsis

**DOI:** 10.1371/journal.pgen.1004956

**Published:** 2015-02-13

**Authors:** Congyao Xu, Xin Zhou, Chi-Kuang Wen

**Affiliations:** National Key Laboratory of Plant Molecular Genetics and National Center for Plant Gene Research (Shanghai), Institute of Plant Physiology and Ecology, Shanghai Institutes for Biological Sciences, Chinese Academy of Sciences, Shanghai, China; Cornell University, UNITED STATES

## Abstract

Arabidopsis REVERSION-TO-ETHYLENE SENSITIVITY1 (RTE1) represses ethylene hormone responses by promoting ethylene receptor ETHYLENE RESPONSE1 (ETR1) signaling, which negatively regulates ethylene responses. To investigate the regulation of RTE1, we performed a genetic screening for mutations that suppress ethylene insensitivity conferred by *RTE1* overexpression in Arabidopsis. We isolated *HYPER RECOMBINATION1* (*HPR1*), which is required for *RTE1 overexpressor* (*RTE1ox*) ethylene insensitivity at the seedling but not adult stage. HPR1 is a component of the THO complex, which, with other proteins, forms the TRanscription EXport (TREX) complex. In yeast, *Drosophila*, and humans, the THO/TREX complex is involved in transcription elongation and nucleocytoplasmic RNA export, but its role in plants is to be fully determined. We investigated how HPR1 is involved in *RTE1ox* ethylene insensitivity in *Arabidopsis*. The *hpr1-5* mutation may affect nucleocytoplasmic mRNA export, as revealed by *in vivo* hybridization of fluorescein-labeled oligo(dT)45 with unidentified mRNA in the nucleus. The *hpr1-5* mutation reduced the total and nuclear *RTE1* transcript levels to a similar extent, and *RTE1* transcript reduction rate was not affected by *hpr1-5* with cordycepin treatment, which prematurely terminates transcription. The defect in the THO-interacting TEX1 protein of TREX but not the mRNA export factor SAC3B also reduced the total and nuclear *RTE1* levels. SERINE-ARGININE-RICH (SR) proteins are involved mRNA splicing, and we found that SR protein SR33 co-localized with HPR1 in nuclear speckles, which agreed with the association of human TREX with the splicing machinery. We reveal a role for HPR1 in *RTE1* expression during transcription elongation and less likely during export. Gene expression involved in ethylene signaling suppression was not reduced by the *hpr1-5* mutation, which indicates selectivity of HPR1 for *RTE1* expression affecting the consequent ethylene response. Thus, components of the THO/TREX complex appear to have specific roles in the transcription or export of selected genes.

## Author Summary

The transcription and export of RNA is a dynamic and highly coordinated process. mRNA species that are selectively mediated by the THO/TRanscription EXport (THO/TREX) complex for their transcription and export remain to be identified. As well, the specific roles of complex components in transcription-coupled export are unclear. We reveal a role for HYPER RECOMBINATION1 (HPR1) [the yeast HYPER RECOMBINATION1 (Hpr1) homolog] in *REVERSION-TO-ETHYLENE SENSITIVITY1* (*RTE1*) transcription elongation but not activation or export, which agrees with the role of yeast Hpr1 in transcription elongation. Defects in the THO/TREX component TEX1 but not the RNA-export TREX-2 component SAC3B also reduced the native *RTE1* level. Our data suggest a specific role of the THO/TREX component HPR1 in *RTE1* expression. Whether TEX1 is involved in *RTE1* transcription or stability remains to be determined. The yeast Sub2 protein is an RNA helicase involved in unwinding the inhibitory structure in the nascent RNA, and *SUB2* overexpression suppresses yeast *Δhpr1* defects; HPR1 could be involved in expression of selected genes with higher-order structure, where RNA polymerase movement could pause. Studies of the gene structure and transcription activity could shed light on roles of these components in gene expression regulation at the transcription-export level.

## Introduction

In Arabidopsis, the gaseous plant hormone ethylene is perceived by a family of five ethylene receptor members that structurally resemble prokaryotic “two-component” histidine kinases (HKs). Ethylene receptor signaling is not associated with HK activity, and the biochemical nature of the receptor signal remains to be determined [1]. Without biochemical knowledge of the ethylene receptor signal output, ethylene receptor signaling is largely evaluated by ethylene-induced growth inhibition or altered gene expression.

Through molecular genetic studies, a model for ethylene receptor signaling has been proposed. Without ethylene, the ethylene receptors mediate the receptor signal output to suppress ethylene signaling; ethylene binding to the receptors prevents receptor signaling and suppression of ethylene signaling is relieved [2, 3]. The five members of the ethylene receptor family have common and divergent functions and act cooperatively as complexes [4–7]. The ethylene receptor ETR1 can function largely independently of other family members to suppress the ethylene response to a great extent. In contrast, ETHYLENE RESPONSE SENSOR1 (ERS1) functions differentially depending on other family members [6, 7].

*REVERSION-TO-ETHYLENE SENSITIVITY1* (*RTE1*) was isolated from a mutation that suppresses the dominant ethylene-insensitive *etr1–2* mutation [8]. *RTE1* encodes a membrane protein that is associated with the endoplasmic reticulum (ER) and Golgi apparatus [9, 10]. *RTE1* overexpression results in ethylene insensitivity in wild-type plants depending on ETR1 ethylene receptor but not on other family members possibly via the ETR1 N-terminus [8, 10–12]. RTE1 homologs are prevalent in higher eukaryotes, and expression of the rice *RTE1 HOMOLOG1* (*OsRTH1*) complements the *rte1–2* loss-of-function mutation and promotes ETR1 receptor signaling in Arabidopsis [13]. Elevated expression of the tomato RTE1 homolog GREEN RIPE (GR), with the dominant *Gr* mutation or *CaMV35S:GR* transgene, results in reduced ethylene responsiveness in fruit tissue and thus the non-ripening phenotype [14]. *RTE1* functions in ethylene signaling may be highly conserved across higher plant species.

To identify components mediating RTE1 functions, we performed a suppressor screen for *RTE1* overexpressor (*RTE1ox*) and isolated *HYPER RECOMBINATION1* (*HPR1*). *HPR1* encodes a protein homologous to yeast (*Saccharomyces cerevisiae*) HYPER-RECOMBINATION1 (Hpr1) protein, a component of the mRNA transcription export machinery, namely the THO complex. Of note, mutations that affect the mRNA transcription export machinery have been reported in plants and they result in various defects in response to pathogens, stresses, and hormones; however, the involved mRNA species are largely undetermined [15–19]. In yeast, the *Δhpr1* mutation results in increased intrachromosomal recombination events that specifically depend on transcription elongation involving RNA polymerase II [20–23]. Proper progression of transcription over specific DNA structures would otherwise pause without Hpr1 [21]. Arabidopsis HPR1 was previously suggested to be involved in *trans*-acting small interfering RNA (tasi-RNA) production [15, 24], and the mutation also confers pleiotropic phenotypes and reduced *ERECTA* expression and defects in alternative splicing [15, 25, 26]. Arabidopsis *hpr1–4* showed bulk nuclear mRNA accumulation, and the mRNA species that requires HPR1 for nucleocytoplasmic export remains to be determined [26].

Yeast THO comprises Hpr1, Mft1, Tho2, and Thp2, and Hpr1 bridges the Sub2-Yra1 dimer to form the THO/TRanscription EXport (TREX) complex [20, 23, 27, 28]. Other proteins, such as TEX1, were identified in yeast, human and Arabidopsis THO/TREX [24]. Transcription is dynamic and involves coordinated and complicated processes including transcription elongation, docking of various RNA binding proteins to the nascent transcript, 5’ capping, 3’ polyadenylation, and mRNA splicing. During transcription, mRNA binding factors and nascent mRNAs are assembled dynamically into the messenger ribonucleaoprotein particles (mRNPs), which are eventually exported through the nuclear pore complex to the cytoplasm. THO/TREX plays an important role in these coordinated processes [27, 29]. Arabidopsis TEX1 is a component of the TREX complex, and defects in TEX1 result in reduced amounts of tasi-RNA species derived from their *TAS1* and *TAS2* precursors but not *TAS3*, which indicates involvement of the THO/TREX complex in the processing or transport of RNA precursors for certain tasi-RNA species [15, 24].

The isolation of *HPR1* prompted us to investigate how *RTE1* functions were affected in the absence of *HPR1*. Results from our study provide evidence supporting the involvement of the THO/TREX component HPR1 in *RTE1* expression and less likely in its degradation and export, which is consistent with the role of yeast Hpr1 in transcription elongation [20, 23]. We also investigated the roles of other components of the transcription-export machinery in *RTE1* expression. Nuclear *RTE1* level was reduced in the absence of TEX1, and the role of TEX1 in *RTE1* expression remains to be determined.

## Results

### Loss-of-function Allele of *SUPPRESSOR OF RTE1 OVEREXPRESSION1* (*SRT1*) Suppresses *RTE1ox*

To isolate components involving *RTE1* functions, we performed a suppressor screen for an *RTE1* overexpressor [*RTE1ox*; *35S:RTE1* expressed in the wild type (Col-0)]. Ethylene treatment inhibited the hypocotyl elongation of etiolated wild-type (Col-0) seedlings. *RTE1ox* was ethylene insensitive, and the seedling produced a long hypocotyl with ethylene treatment [8, 10]. We isolated the mutagenized *RTE1ox* seedlings, at the M2 generation, showing ethylene growth inhibition. Genes required for ethylene insensitivity conferred by *RTE1* overexpression were designated *SRT*.

**Figure 1:**
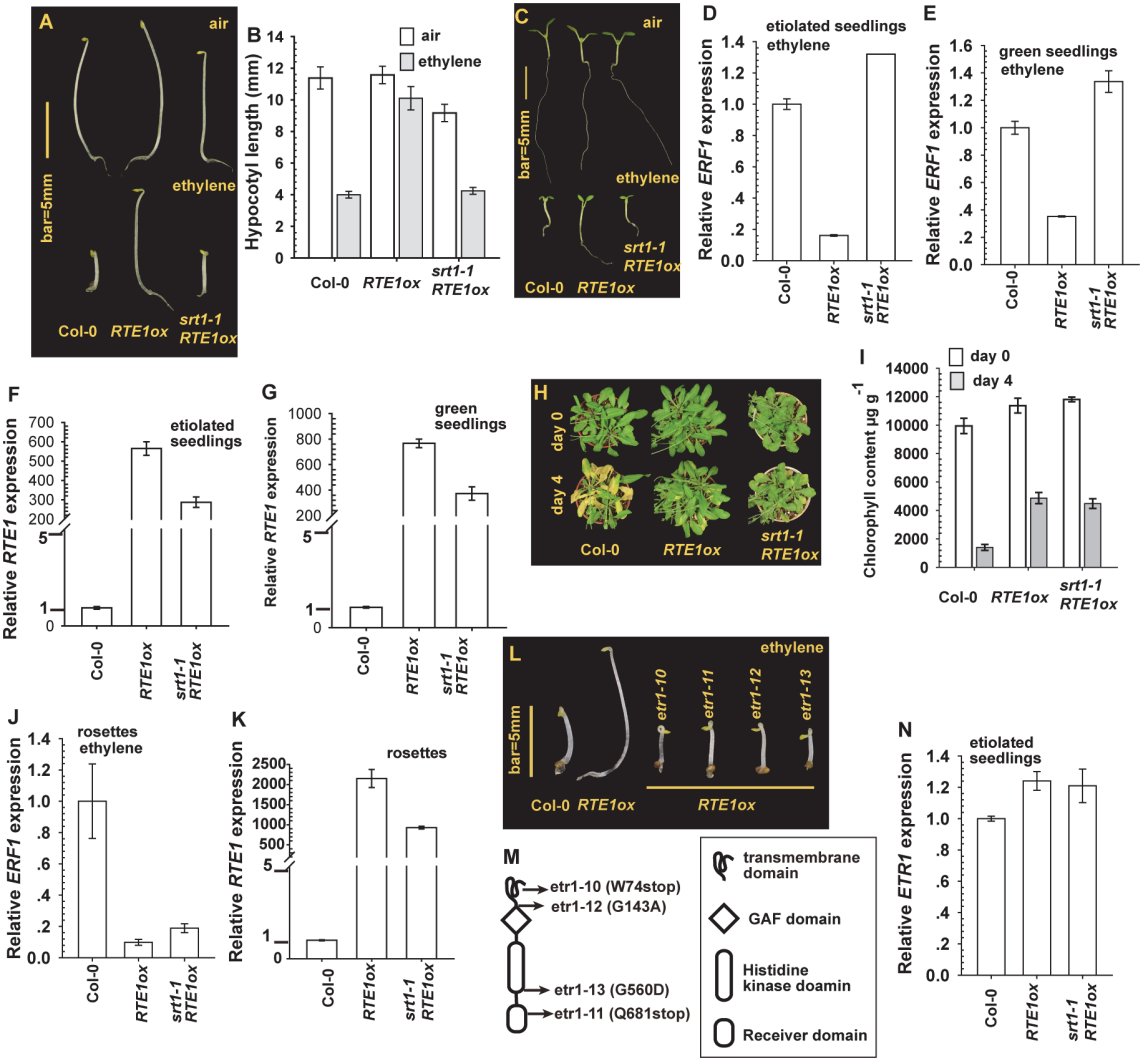
Effect of *SUPPRESSOR OF RTE1 OVEREXPRESSION1* (*SRT1*) on the ethylene insensitivity in *REVERSION-TO-ETHYLENE SENSITIVITY1 overexpressor* (*RTE1ox*). Etiolated seedling phenotype (A), hypocotyl measurement (B), and light-grown seedling phenotype (C) for *RTE1ox* and *srt1–1 RTE1ox* seedlings. Expression of *ETHYLENE RESPONSE FACTOR1* (*ERF1*) in *RTE1ox* with the *srt1–1* allele in etiolated (D) and light-grown (E) seedlings on ethylene treatment. *RTE1* expression in *RTE1ox* and *srt1–1 RTE1ox* seedlings grown in dark (F) and light (G). Leaf senescence phenotype (H) and chlorophyll content (I) in *RTE1ox* and *srt1–1 RTE1ox* plants. *ERF1* (J) and *RTE1* (K) expression in rosette leaves. Phenotype (L) of etiolated *RTE1ox* seedlings with the indicated *etr1* mutations (M). (N) *ETR1* expression is not altered by *srt1–1*. Data are mean±SD for seedling hypocotyls (n>30) and chlorophyll content (n>3), and mean± SE for gene expression (at least 3 independent biological samples with 3 measurement for each). Ethylene concentration is 10 μL L^-1^ for the seedling growth inhibition test and *ERF1* induction, and 100 μL L^-1^ for the leaf senescence test.

Etiolated seedlings of the wild type (Col-0), *RTE1ox*, and *srt1–1 RTE1ox* produced a long hypocotyl without ethylene treatment; ethylene treatment inhibited the hypocotyl elongation of wild-type and *srt1–1 RTE1ox* but not *RTE1ox* seedlings (Fig. 1A). With ethylene treatment, the hypocotyl was short for wild-type and *srt1–1 RTE1ox* seedlings and relatively long for *RTE1ox* seedlings (Fig. 1B).

Germinated under light, ethylene-treated wild-type and *srt1–1 RTE1ox* seedlings showed growth inhibition, with small and compact cotyledons and a short root, whereas *RTE1ox* seedlings produced expanded cotyledons and a long root (Fig. 1C). Without ethylene treatment, all genotypes examined showed a normal growth phenotype. The expression of *ETHYLENE RESPONSE FACTOR1* (*ERF1*) is induced by ethylene treatment [30, 31]; with ethylene, *ERF1* level was higher in wild-type and *srt1–1 RTE1ox* than *RTE1ox* seedlings (Fig. 1D-1E). The suppression of *RTE1ox* by *srt1–1* may be associated with reduced *RTE1* level at the seedling stage (Fig. 1F-1G).

At the adult stage, ethylene did not promote leaf senescence in ethylene-insensitive plants. With 4-day ethylene treatment, wild-type but not *RTE1ox* and *srt1–1 RTE1ox* plants showed the leaf senescence phenotype (Fig. 1H). Consistently, after ethylene treatment, the chlorophyll content was more greatly reduced in the wild type (Col-0) than in *RTE1ox* and *srt1–1 RTE1ox* plants (Fig. 1I). Ethylene treatment elevated the *ERF1* level in rosette leaves of the wild type but not *RTE1ox* and *srt1–1 RTE1ox* leaves (Fig. 1J), although *RTE1* level was reduced by the *srt1–1* allele (Fig. 1K).

Of note, in the suppressor screen, 4 alleles of *ETR1* ethylene receptor gene were isolated (Fig. 1L-1M), consistent with the notion of ethylene insensitivity conferred by *RTE1* overexpression depending on ETR1 [8, 10, 32]. Among these alleles, *etr1–10* was previously described with the W74stop early termination [33]. *ETR1* expression was not altered by the *srt1–1* mutation; *srt1–1* did not reduce *ETR1* level to suppress *RTE1ox* (Fig. 1N).

The *srt1–1* allele prevented ethylene insensitivity conferred by *RTE1* overexpression mainly during the seedling but not adult stage. In *RTE1ox*, *RTE1* level was reduced by the *srt1–1* allele throughout development. A lesser amount of RTE1 could be sufficient to confer ethylene insensitivity at the adult but not seedling stage.

### Cloning of *SRT1* and Isolation of the Loss-of-function Alleles

**Figure 2:**
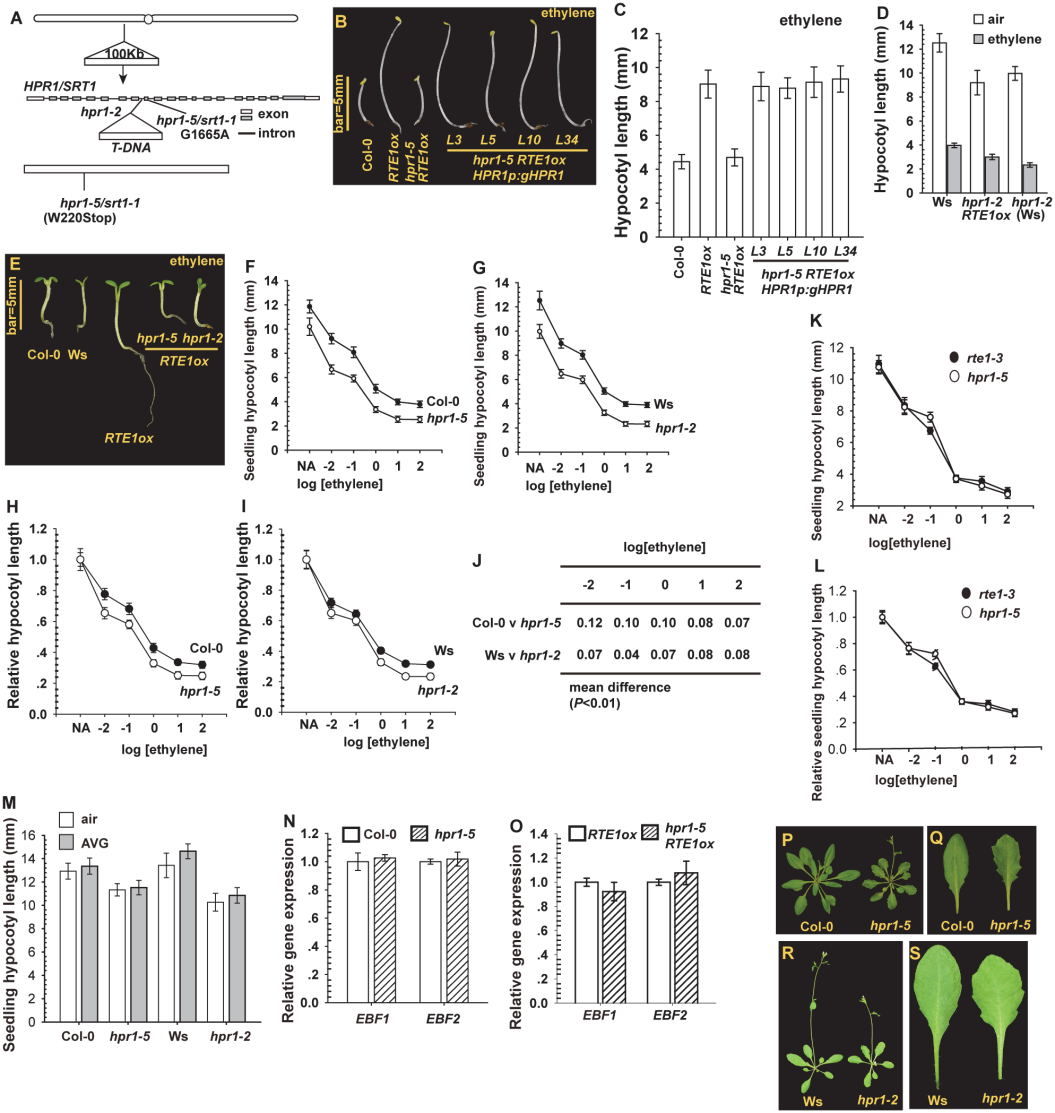
The cloning and isolation of *HPR1*/*SRT1* and the mutants. (A) Map-based cloning of *HPR1/SRT1*, and isolation of the *hpr1–5*/*srt1–1* and *hpr1–2* alleles. Etiolated seedling phenotype (B) and hypocotyl measurement (C) for *hpr1–5 RTE1ox* expressing the *HPR1p:gHPR1* transgene. Etiolated seedling hypocotyl measurement (D) and light-grown seedling phenotype (E) for *hpr1–2 RTE1ox*. Ethylene concentration is 10 μL L^-1^. Ethylene dose–response assay by etiolated seedling hypocotyl measurement of *hpr1–5* (F) and *hpr1–2* (G) and by normalized hypocotyl measurement for *hpr1–5* (H) and *hpr1–2* (I). (J) Mean difference in normalized hypocotyl length between wild-type and *hpr1–5* and *hpr1–2* seedlings. Ethylene dose-response curves of the absolute (K) and normalized (L) lengths of hypocotyls of etiolated *rte1–3* and *hpr1–5* seedlings. (M) Hypocotyl length of etiolated seedlings with AVG treatment. Relative gene expression of *EIN3-BINDING F-BOX PROTEIN1* (*EBF1*) and *EBF2* in the wild type (Col-0) and *hpr1–5* (N) and *RTE1ox* and *hpr1–5 RTE1ox* (O). Rosette (P) and leaf (Q) phenotype of the wild type (Col-0) and *hpr1–5* plants. Rosette (R) and leaf (S) phenotype of the wild type (Ws) and *hpr1–2* plants. Data are mean±SD for seedling hypocotyls (n>30) and mean±SE for gene expression measurement (n = 3 with 3 technical repeats). Student’s *t* test is for paired comparison for means of 2 measurements. Ethylene concentration presented in logarithm of ethylene (μL L^-1^) for the dose-response assay.

We performed map-based cloning and mapped *SRT1* to a 100-kb region at chromosome 5. This region was sequenced, and we identified only one nucleotide change (the G1665A transition mutation), which resulted in the W220stop early termination of the HPR1 protein (Fig. 2A; locus At5g09860); herein *SRT1* is designated *HPR1* and the *srt1–1* allele *hpr1–5*. A suppressor screen of the loss-of-function mutant of *ENHANCED DISEASE RESISTANCE1* (*EDR1*) isolated a loss-of-function allele of *HPR1* [26] at the *ERECTA mRNA UNDER-EXPRESSED* (*EMU*) locus, and the loss-of-function *emu* mutant produced a reduced amount of *ERECTA* and several miRNAs [25]. Sequence analysis suggested that HPR1/EMU shared relatively low homology with the yeast (*Saccharomyces cerevisiae*) Hpr1 protein of the THO/TREX complex involved in mRNA transcription and export [25]. A genomic clone for *HPR1* (*HPR1p:gHPR1*) was introduced into *hpr1–5 RTE1ox* by transformation, and etiolated seedlings of the resulting lines were ethylene-insensitive and produced a long hypocotyl with ethylene treatment (Fig. 2B-2C). The *hpr1–2* mutation [in a Wassilewskija (Ws) ecotype background] resulted from a *T*-DNA insertion at the 8^th^ intron of *HPR1* [15]; etiolated *hpr1–2 RTE1ox* seedlings also showed growth inhibition with ethylene (Fig. 2A and 2D). Consistently, ethylene treatment inhibited the growth of light-grown *hpr1–5 RTE1ox* and *hpr1–2 RTE1ox* seedlings (Fig. 2E).

Of note, *hpr1–5* and *hpr1–2* single mutants produced a slightly shorter seedling hypocotyl than did wild-type seedlings over a wide range of ethylene concentration (Fig. 2F-2G). The seedling hypocotyl length was measured and normalized to that of non-treated seedlings; the relative seedling hypocotyl lengths were slightly shorter for *hpr1–5* and *hpr1–2* than the wild type (Col-0 and Ws) (Fig. 2H-2J).

*RTE1ox* was suppressed by *hpr1–5*, which prompted us to evaluate whether *RTE1* function could be suppressed by *hpr1–5* such that *hpr1* and *rte1* seedlings could behave similarly in response to ethylene treatment. Over a wide range of ethylene concentration, *hpr1–5* and the loss-of-function *rte1–3* seedlings produced a nearly identical dose-response curve for seedling hypocotyl length (Fig. 2K–2L). On treatment with the ethylene biosynthesis inhibitor aminoethoxyvinylglycine (AVG), *hpr1* seedling hypocotyl growth was not rescued to the wild-type level, so the seedling hypocotyl shortening was not due to an elevation in endogenous ethylene biosynthesis (Fig. 2M).

The F-box proteins EIN3-BINDING F-BOX PROTEIN1 (EBF1) and EBF2 have a role in EIN3 degradation via the 26S proteasome, and the mutants are hypersensitive to ethylene, with a weak phenotype of seedling growth inhibition [34, 35]. We investigated whether the *hpr1–5* mutation could affect *EBF1* and *EBF2* expression and therefore seedling growth. qRT-PCR revealed nearly identical expression of *EBF1* and *EBF2* between wild-type (Col-0) and *hpr1–5* seedlings, regardless of the *35S:RTE1* transgene (Fig. 2N-2O). *RTE1ox* suppression and increased ethylene sensitivity by the *hpr1–5* allele was not associated with *EBF1/EBF2* levels.

At the adult stage, *hpr1–5* and *hpr1–2* plants were smaller than their wild-type ecotypes (Col-0 and Ws) in rosette size, and mutant leaves were serrated (Fig. 2P-2S), which is consistent with the *emu* mutant phenotype [25]. *HPR1* could have roles in leaf morphogenesis and other aspects of plant growth and development in addition to its role in *RTE1* level.

### *hpr1–5* Inhibits Ethylene Insensitivity Conferred by *etr1–2* but not *ein2–50* and *ein3–1*

The signal output by ETR1 that inhibits ethylene signaling is prevented by ethylene. The *etr1–2* mutation, resulting from an A102T substitution, confers dominant ethylene insensitivity [36]. *RTE1* is essential to ethylene insensitivity conferred by *etr1–2* [8, 10]. Given that *hpr1–5* suppressed *RTE1ox* and that *hpr1–5* and *rte1–3* seedlings behaved similarly in response to ethylene treatment (Fig. 2K–2L), we wondered whether the *hpr1–5* mutation could also suppress *etr1–2*.

**Figure 3:**
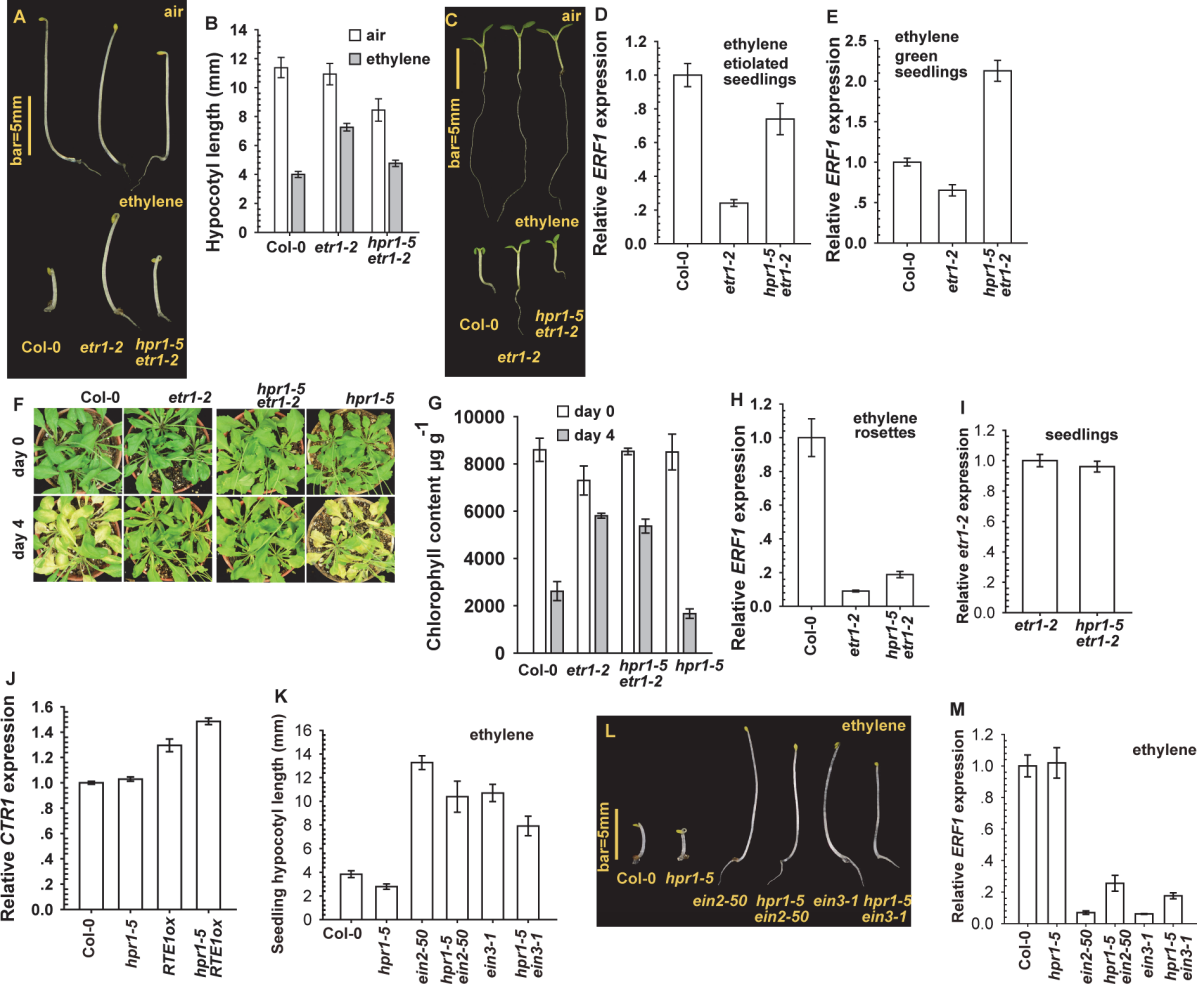
The *hpr1–5* allele suppressed *etr1–2* ethylene insensitivity at the seedling stage. Etiolated seedling phenotype (A), hypocotyl measurement (B), and light-grown seedling phenotype (C) for *etr1–2* and *hpr1–5 etr1–2*. *ERF1* expression in etiolated (D) and light-grown (E) seedlings of *etr1–2* and *hpr1–5 etr1–2*. Leaf senescence phenotype (F) and chlorophyll content (G) in *etr1–2* and *hpr1–5 etr1–2* plants. (H) *ERF1* expression in *etr1–2* and *hpr1–5 etr1–2* rosettes. Expression of *etr1–2* (I) and *CTR1* (J) is not reduced by the *hpr1–5* mutation. Measurement (K) and phenotype (L) of hypocotyls of etiolated seedlings of *ein2–50* and *ein3–1* mutants with and without the *hpr1–5* allele, and *ERF1* expression in these genotypes (M). Data are mean±SD seedling hypocotyls (n>30) and chlorophyll content (n>3), and mean±SE for gene expression (at least 3 independent biological samples with 3 measurements for each). Ethylene concentrations are 10 μL L^-1^ for *ERF1* induction and 100 μL L^-1^ for the leaf senescence test.

With ethylene treatment, etiolated *hpr1–5 etr1–2* but not *etr1–2* seedlings showed growth inhibition (Fig. 3A), which agrees with the seedling hypocotyl measurement (Fig. 3B). Consistently, ethylene growth inhibition was stronger for light-grown wild-type (Col-0) and *hpr1–5 etr1–2* than *etr1–2* seedlings (Fig. 3C). With ethylene treatment, *ERF1* level was high for wild-type and *hpr1–5 etr1–2* seedlings and low for *etr1–2* seedlings (Fig. 3D-3E).

The *hpr1–5* allele had little effect on the ethylene insensitivity conferred by *RTE1* overexpression at the adult stage (Fig. 1H-1J). As expected, the *hpr1–5* allele had little effect on *etr1–2* at the adult stage, and 4-day ethylene treatment did not induce leaf senescence in *etr1–2* or *hpr1–5 etr1–2* rosette leaves (Fig. 3F). Reduction in chlorophyll content was greater in wild-type (Col-0) and *hpr1–5* than *etr1–2* and *hpr1–5 etr1–2* plants (Fig. 3G). Consistently, *ERF1* level that was elevated in the wild type (Col-0) was not elevated with ethylene treatment in *etr1–2* or *hpr1–5 etr1–2* adult plants (Fig. 3H). qRT-PCR revealed a similar *etr1–2* expression in *hpr1–5 etr1–2* and *etr1–2* seedlings, so *hpr1–5* did not affect *etr1–2* level to suppress *etr1–2* ethylene insensitivity (Fig. 3I). Of note, CONSTITUTIVE TRIPLE RESPONSE1 (CTR1) is a Raf-like protein mediating the ethylene receptor signaling to repress ethylene signaling, and defects in CTR1 result in elevated ethylene responses [37, 38]. *CTR1* expression was not reduced by *hpr1–5* (Fig. 3J), so the *hpr1–5* mutation did not impact *CTR1* level to affect the ethylene signaling.

We considered whether *hpr1–5* could simply suppress ethylene insensitivity without specificity for *RTE1*. This scenario was evaluated by examining whether ethylene insensitivity by other mutations in the ethylene signaling pathway could be suppressed by *hpr1–5*. ETHYLENE INSENSITIVE2 (EIN2) acts downstream of CTR1 to mediate ethylene signaling to the transcription factor EIN3 for inducing the ethylene response [39]. The loss-of-function *ein2–50* (from T-DNA insertion) [40, 41] and *ein3–1* [30] mutants were ethylene-insensitive and each was crossed with *hpr1–5* to obtain *hpr1–5 ein2–50* and *hpr1–5 ein3–1*. With ethylene treatment, etiolated *ein2–50* and *ein3–1* seedlings were ethylene-insensitive and the hypocotyl elongation was not inhibited, whereas wild-type (Col-0) and *hpr1–5* seedlings were ethylene-responsive, with hypocotyl growth inhibition (Fig. 3K-3L). Seedlings of *hpr1–5 ein2–50* and *hpr1–5 ein3–1* were slightly shorter than those of *ein2–50* and *ein3–1*, respectively, possibly because of the intrinsic shortness of *hpr1–5* seedlings. Consistently, *ERF1* expression was relatively low in genotype with *ein2–50* or *ein3–1* compared to the wild type (Col-0) and *hpr1–5* (Fig. 3M).

The suppression of *etr1–2* seedling ethylene insensitivity by *hpr1–5* agreed with the suppression of *RTE1ox* ethylene insensitivity by *hpr1–5*, which supports *HPR1* as essential to *RTE1* functions at the seedling stage. With the inability of *hpr1–5* to suppress ethylene insensitivity of the *ein2–50* and *ein3–1* mutations, the suppression of *etr1–2* and *RTE1* functions by *hpr1–5* was not achieved by inhibiting ethylene insensitivity, which indicates specificity of *HPR1* for *RTE1*.

### The *hpr1–5* Allele Prevents Export of Bulk mRNA but Not *RTE1* Transcripts

HPR1 shares homology with yeast Hpr1, so HPR1 may have a role in mRNA processing and export from the nucleus to the cytoplasm. The role of Arabidopsis HPR1 in bulk mRNA export was recently demonstrated in the *hpr1–4* allele [26]. We examined whether the suppression of *RTE1ox* and *etr1–2* mutation by the *hpr1–5* allele was due to impaired *RTE1* transcript export.

**Figure 4:**
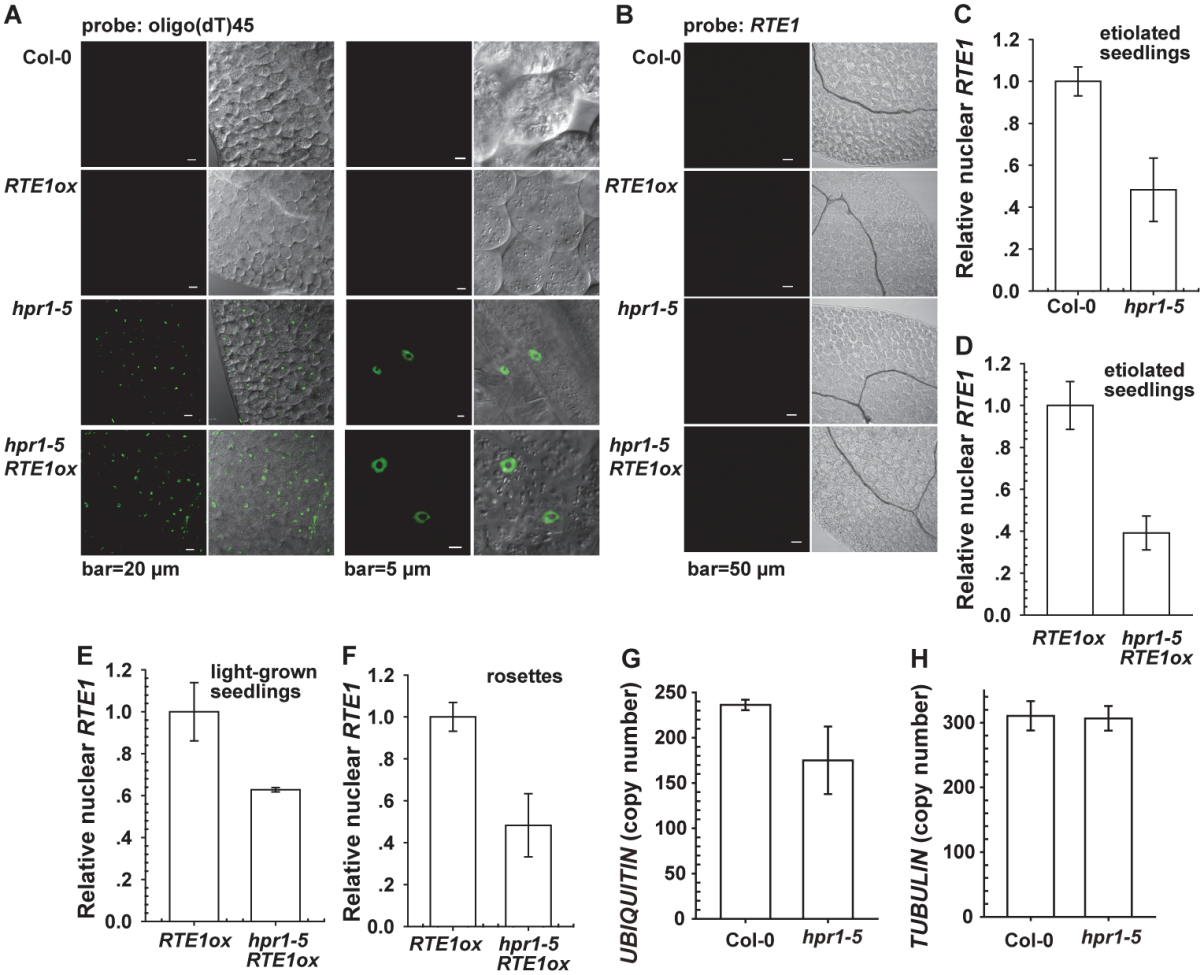
Effect of the *hpr1–5* allele on bulk and *RTE1* mRNA accumulation in the nucleus. Nuclear fluorescence by fluorescine-labeled oligo(dT)_45_ (A) and *RTE1* (B) probes. Nuclear *RTE1* level in etiolated seedlings of the wild-type (Col-0) and *hpr1–5* (C); etiolated (D) and light-grown (E) *RTE1ox* and *hpr1–5 RTE1ox* seedlings; and *RTE1ox* and *hpr1–5 RTE1ox* rosette leaves (F). Measurement of the nuclear *UBIQUITIN* (G) and *TUBULIN* (H) transcript copy number in the wild type (Col-0) and *hpr1–5*. Data are mean±SE, with 3 measurements for each 3 independent biological samples.

With the fluoresceine-labeled oligo(dT)_45_ as a probe hybridized with polyadenylated mRNA, *hpr1–5* and *hpr1–5 RTE1ox* but not wild-type or *RTE1ox* cells produced fluorescence within the nucleus (Fig. 4A). The results suggest that the suppression of *RTE1ox* by *hpr1–5* could be associated with impaired bulk mRNA export. The impaired export of mRNA species that involve RTE1 functions could result in the suppression of *RTE1ox* and *etr1–2*. Alternatively, HPR1 may directly involve *RTE1* transcription or export, and the *hpr1–5* mutation suppressed *RTE1ox* and *etr1–2*. Therefore, we examined whether *RTE1* transcripts could accumulate in the nucleus of the *hpr1–5* mutant; none of those genotypes produced fluorescence in the nucleus when hybridized with the fluoresceine-labeled *RTE1* probe (Fig. 4B).

The lack of evidence for *RTE1* transcript accumulation in the nucleus of the *hpr1–5* mutant could be due to limited detection sensitivity or *hpr1–5* not affecting *RTE1* transcript export. Therefore, we measured nuclear *RTE1* levels in the wild type (Col-0) and genotypes with the *hpr1–5* alleles. Nuclei were isolated from the wild type, *RTE1ox*, *hpr1–5*, and *hpr1–5 RTE1ox* for RNA isolation. The nuclear *RTE1* level was reduced to 40% to 60% by the *hpr1–5* allele (Fig. 4C- 4F). The total (Fig. 1F, 1G and 1K) and nuclear *RTE1* level (Fig. 4C-4F) was reduced to a similar level by the *hpr1–5* allele, with no nuclear *RTE1* accumulation (Fig. 4B). Therefore, *hpr1–5* had little effect on *RTE1* transcript export. Of note, considering that defects caused by the *HPR1* mutation could affect expression of the calibration gene on qRT-PCR, we examined the expression of 2 widely used normalization genes. By calibrating against the standard curve, with cDNA used as the template, the copy number for the nuclear *UBIQUITIN* was reduced in *hpr1–5* compared to the wild type (Col-0) (Fig. 4G) and unaltered for the nuclear *TUBULIN* (Fig. 4H). Throughout the work, if not specified, *TUBULIN* was used as the normalization gene for qRT-PCR.

*HPR1/EMU* is required for normal expression of *ERECTA* and several miRNAs [25]. Our results showed that *RTE1* level was reduced in the *hpr1–5* mutant; these data support HPR1 as required for the normal expression of *RTE1*.

### *RTE1* Transcript Degradation Is Not Accelerated by the *hpr1–5* Allele

The nuclear *RTE1* transcription was reduced by the *hpr1–5* allele, which could be caused by reduced transcription activity or accelerated degradation. Cordycepin (or 3'-deoxyadenosine) is structurally analogous to adenosine, and its incorporation into RNA molecules causes premature termination of biosynthesis. Treatment with cordycepin to terminate transcription facilitates kinetics study of the transcript degradation [42–45]. *eIF4A* has a prolonged, stable half life [46] and was used as an ideal internal reference for measuring *RTE1* level with cordycepin treatment.

**Figure 5:**
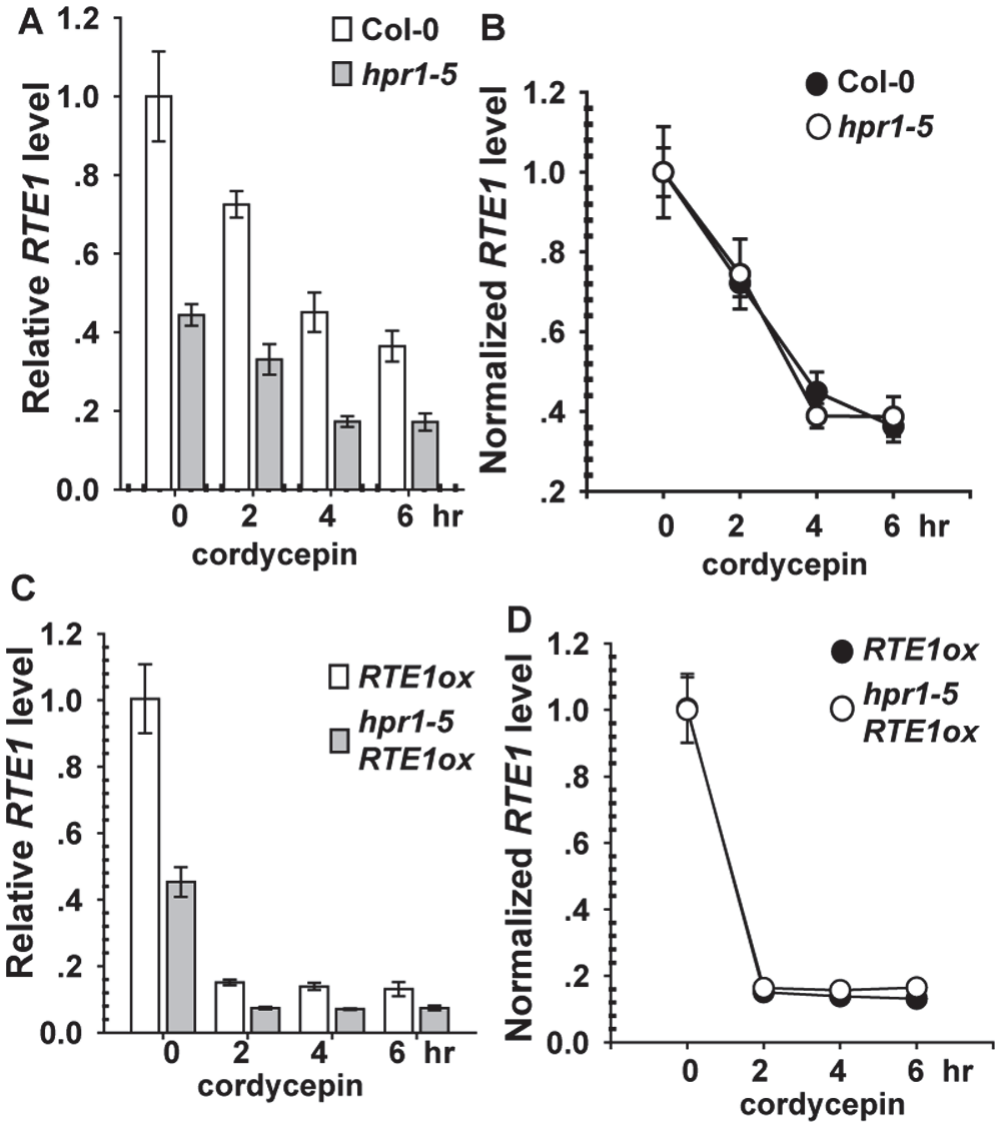
*RTE1* degradation with cordycepin treatment. Measured (A) and normalized (B) *RTE1* level with cordycepin treatment in wild-type (Col-0) and *hpr1–5* seedlings. Measured (C) and normalized (D) *RTE1* level with cordycepin treatment in *RTE1ox* and *hpr1–5 RTE1ox* seedlings. Data are mean±SE, with 3 measurements for each 3 independent biological samples.

Light-grown seedlings of Col-0, *hpr1–5*, *RTE1ox*, and *hpr1–5 RTE1ox* were incubated with cordycepin to terminate RNA biosynthesis. As compared with before cordycepin treatment (time 0), after treatment, *RTE1* level was reduced over time, with a lower level for *hpr1–5* than wild-type seedlings (Fig. 5A). To evaluate the degradation rate, *RTE1* levels were normalized to the level at time 0 (before treatment); normalized levels were identical in the wild type and *hpr1–5* (Fig. 5B). Consistently, *RTE1* level in *RTE1ox* and *hpr1–5 RTE1ox* decreased quickly after treatment, with a greater level for *RTE1ox* than *hpr1–5 RTE1ox* (Fig. 5C). When normalized to the level at time 0, *RTE1* levels were identical in *RTE1ox* and *hpr1–5 RTE1ox* (Fig. 5D). Therefore, the *hpr1–5* allele did not accelerate *RTE1* degradation, so *hpr1–5* could possibly affect *RTE1* transcription.

### HPR1 Is a Nuclear Protein Co-localized with mRNA Splicing Complex

HPR1 was predicted to be a yeast Hpr1 homolog involved in RNA transcription and export. To further our knowledge of HPR1 function, we studied its subcellular localization with the expression of GREEN FLUORESCENT PROTEIN (GFP)-tagged HPR1.

**Figure 6:**
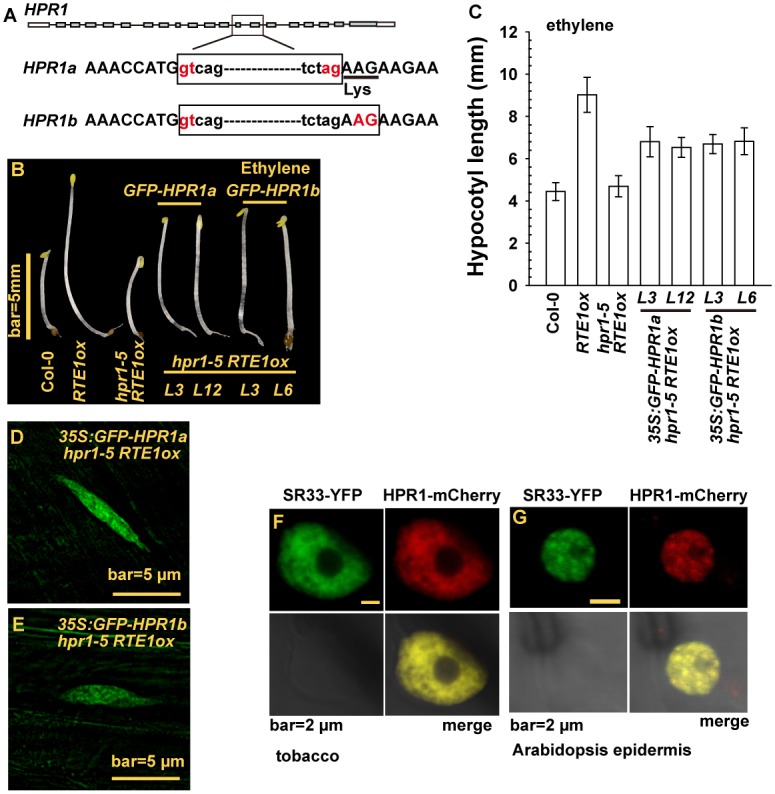
*HPR1* produces 2 transcripts and HPR1 subcellular localization. (A) *HPR1* produces 2 transcripts by alternative splicing. Phenotype (B) and hypocotyl measurement (C) of etiolated *hpr1–5 RTE1ox* seedlings expressing *GREEN FLUORESCENT PROTEIN* (*GFP*)-fused *HPR1a* and *HPR1b*. The fluorescence of GFP-HPR1a (D), GFP-HPR1b (E) in *hpr1–5 RTE1ox* seedlings. Speckle domain co-localization of the fluorescence by SR33-YFP and HPR1-mCherry in tobacco (F) and Arabidopsis epidermis (G).

To obtain an *HPR1* cDNA fragment to be fused with *GFP*, we cloned *HPR1* cDNA and could clone 2 *HPR1* cDNA species, *HPR1a* and *HPR1b*. Sequence analysis for the 2 cDNA clones suggested that they resulted from alternative splicing, and *HPR1a* contained an extra codon for lysine. Thus, HPR1a was 1 amino acid residue longer than HPR1b (Fig. 6A). To determine whether either of the HPR1 species could be functional, we fused cDNA clones for *HPR1a* and *HPR1b* with *GFP* and expressed them under regulation of the constitutive *35S* promoter in *hpr1–5 RTE1ox*. With ethylene treatment, etiolated seedlings of *hpr1–5 RTE1ox* were short and those of *RTE1ox* were long. Expression of *35S:GFP-HPR1a* and- *HPR1b* rescued the ethylene growth inhibition phenotype to a great extent in *hpr1–5 RTE1ox*, which agrees with the seedling hypocotyl measurement (Fig. 6B-6C). GFP-HPR1a and-HPR1b both produced fluorescence in the nucleus with a pattern characteristic of nuclear speckle domains in cells of etiolated seedling hypocotyls (Fig. 6D-6E).

THO/TREX is involved in mRNA processing, and human THO co-localizes with splicing factors in nuclear speckle domains [47–49]. We examined whether HPR1 could co-localize with mRNA-splicing proteins. SERINE-ARGININE-RICH (SR) proteins have essential roles in mRNA splicing, and SR33 is a member of the SR protein family involved in pre-mRNA splicing [50]. When co-expressed in tobacco and Arabidopsis epidermis by *Agrobacterium* infiltration, the proteins SR33-YFP and HPR1-mCherry (encoded by a genomic *HPR1* clone fused with *mCherry*) produced fluorescence with an identical speckle pattern in the nucleus but not nucleolus (Fig. 6F-6G). We tried to estimate the relative abundance of *HPR1a* and *HPR1b*. On sequencing 56 independent cDNA clones for *HPR1*, 27 were *HPR1a* and 29 were *HPR1b*, for a 1:1 ratio (χ2 = 0.62, *P* = 0.77).

Thus, *HPR1* may encode 2 proteins, each involved in *RTE1* function. Whether the 2 proteins could act coordinately, independently, or synergistically remains to be investigated. The co-localization of HPR1-mCherry and SR33-YFP provides evidence that in Arabidopsis the THO/TREX complex could co-localize with splicing factors in nuclear speckle domains involving mRNA processing, as observed in human cells [47, 49]. This argument agrees with the *HPR1* allele *emu* producing splicing defects [25].

### RTE1 Protein Level Is Reduced in the *hpr1–5* Mutant

The *hpr1–5* mutation resulted in reduced *RTE1* level and, likely, the protein amount required for promoting ETR1 ethylene receptor signaling. We examined RTE1 protein levels in the *hpr1–5* mutant.

**Figure 7:**
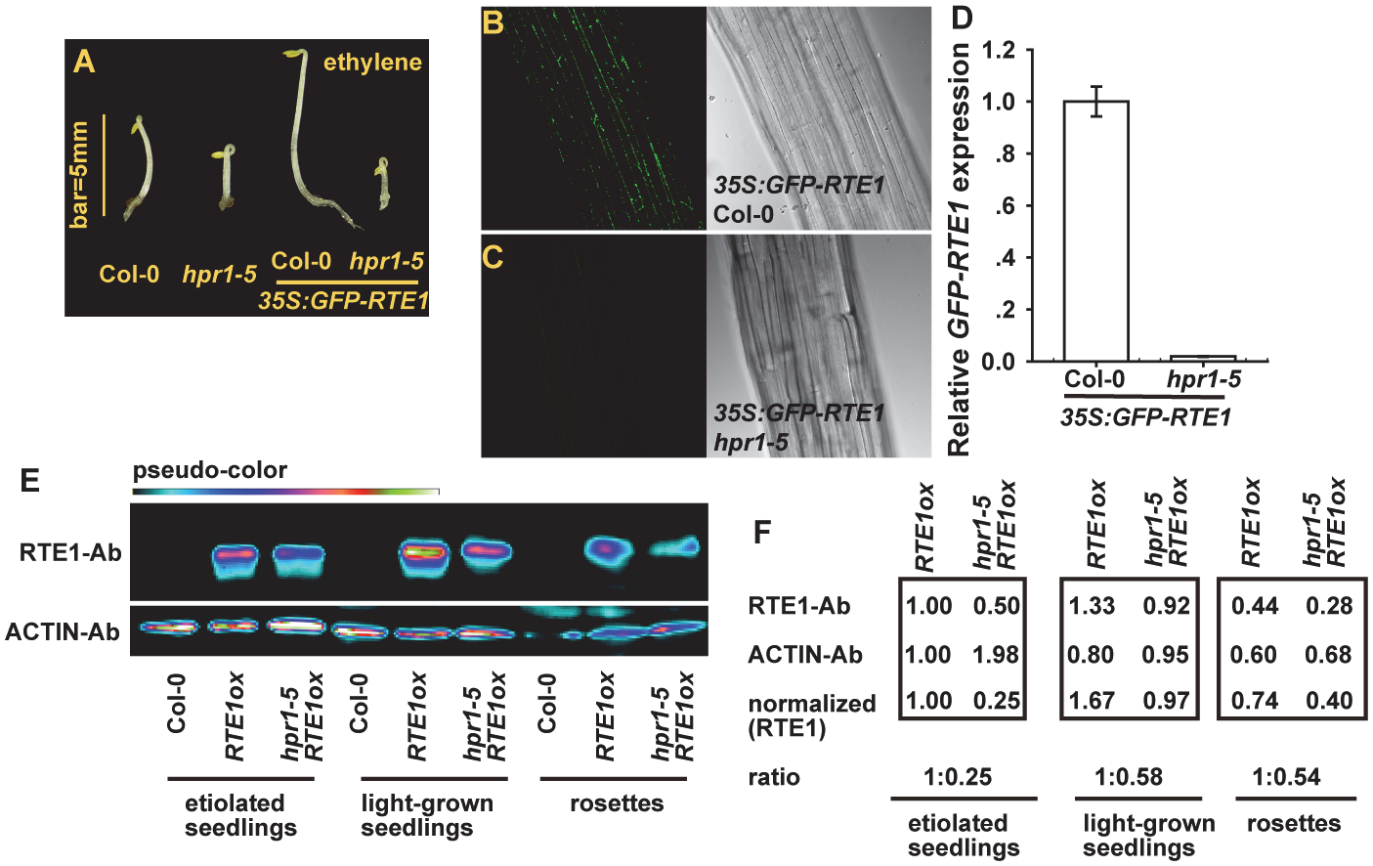
RTE1 level is reduced in the *hpr1–5* mutant. (A) Phenotype of etiolated seedlings of the *hpr1–5* mutant with and without *35S:GFP-RTE1*. Fluorescence of GFP-RTE1 in wild-type (Col-0) (B) and *hpr1–5* (C) seedlings expressing the *35S:GFP-RTE1* transgene and (D) *GFP-RTE1* expression measurement. Data are mean±SE, with 3 measurements for each 3 independent biological samples. Immunofluorescence (E) and quantification (F) of RTE1 level in *RTE1ox* and *hpr1–5 RTE1ox* at seedling and rosette stages. ACTIN was an internal reference for RTE1 normalization. Pseudo-color indicates immunofluorescence of weak (dark) to strong (bright) signal. RTE1 antibodies (RTE1-Ab) and ACTIN-Ab are monoclonal Abs.

A GFP-fused RTE1 was previously shown to be localized mainly at the Golgi apparatus [10]. We found that the expression of *35S:GFP-RTE1* conferred ethylene insensitivity in wild-type (Col-0) but not *hpr1–5* seedlings, and ethylene-treated etiolated seedlings were longer for *35S:GFP-RTE1* (Col-0) than *35S:GFP-RTE1 hpr1–5* (Fig. 7A). Of note, the *35S:GFP-RTE1* transgene in the wild type was introduced into the *hpr1–5* mutant by genetic crossing. The transgene was thus expressed at the same locus in the 2 genotypes, which facilitated study of their expression. We evaluated the association of GFP-RTE1 level and ethylene insensitivity. GFP-RTE1 fluorescence was evident in *35S:GFP-RTE1* (Col-0) seedlings (Fig. 7B) and not detectable in *35S:GFP-RTE1 hpr1–5* seedlings (Fig. 7C). Consistently, relative *GFP-RTE1* level was greatly reduced in *35S:GFP-RTE1 hpr1–5* as compared with *35S:GFP-RTE1* (Col-0) (Fig. 7D).

We considered that the GFP-fused RTE1 was an artificially created protein and its accumulation may not necessarily reflect the status of the native RTE1 protein. Immunoassay for RTE1 amount could help address the effect of the *hpr1–5* allele on RTE1 level. With a monoclonal antibody against RTE1, we observed a strong immune signal for proteins isolated from *RTE1ox*. Unfortunately, sensitivity to detect endogenous RTE1 was poor, and no immune signal could be detected for the protein from the wild type (Col-0). As expected, the RTE1 level in *hpr1–5 RTE1ox* seedlings was reduced throughout development (Fig. 7E). With ACTIN as an internal reference, the amount of RTE1 level was normalized to *RTE1ox* level; relative RTE1 protein level in *RTE1ox* was reduced to 25% to 58% by the *hpr1–5* allele (Fig. 7F).

Our results showed reduced RTE1 protein level by the *hpr1–5* allele, which indicates the basis for why ethylene insensitivity conferred by *RTE1ox* and possibly *etr1–2* was suppressed by the mutation.

### Effect of *sac3b-2* and *tex1–4* Mutations on *RTE1ox*

In yeast (*Saccharomyces cerevisiae*), THO (a tetrameric protein complex comprised of Mft1, Tho2, Thp2, and Hpr1) is linked to the Sub2-Yra1 dimer via Hpr1 to form the TREX complex [20, 49, 51]. TEX1 interacts with THO and is a component of the THO/TREX complex; Arabidopsis *tex1* mutations result in defects in *trans*-acting small interfering RNA (tasi-RNA) production [15, 24]. Sac3 is an mRNA export factor in yeast, constituting the TREX-2 complex with Thp1, Sus1 and Cdc31 for RNA export through the nuclear pore complex (NPC). In addition, RNA transcription elongation and export by THO/TREX is coupled to the NPC-bound TREX-2 complex [16, 27]. With impact of the *hpr1–5* mutation on *RTE1* transcription, we wondered whether other components of the dynamic mRNA transcription export are also involved in *RTE1* expression.

**Figure 8:**
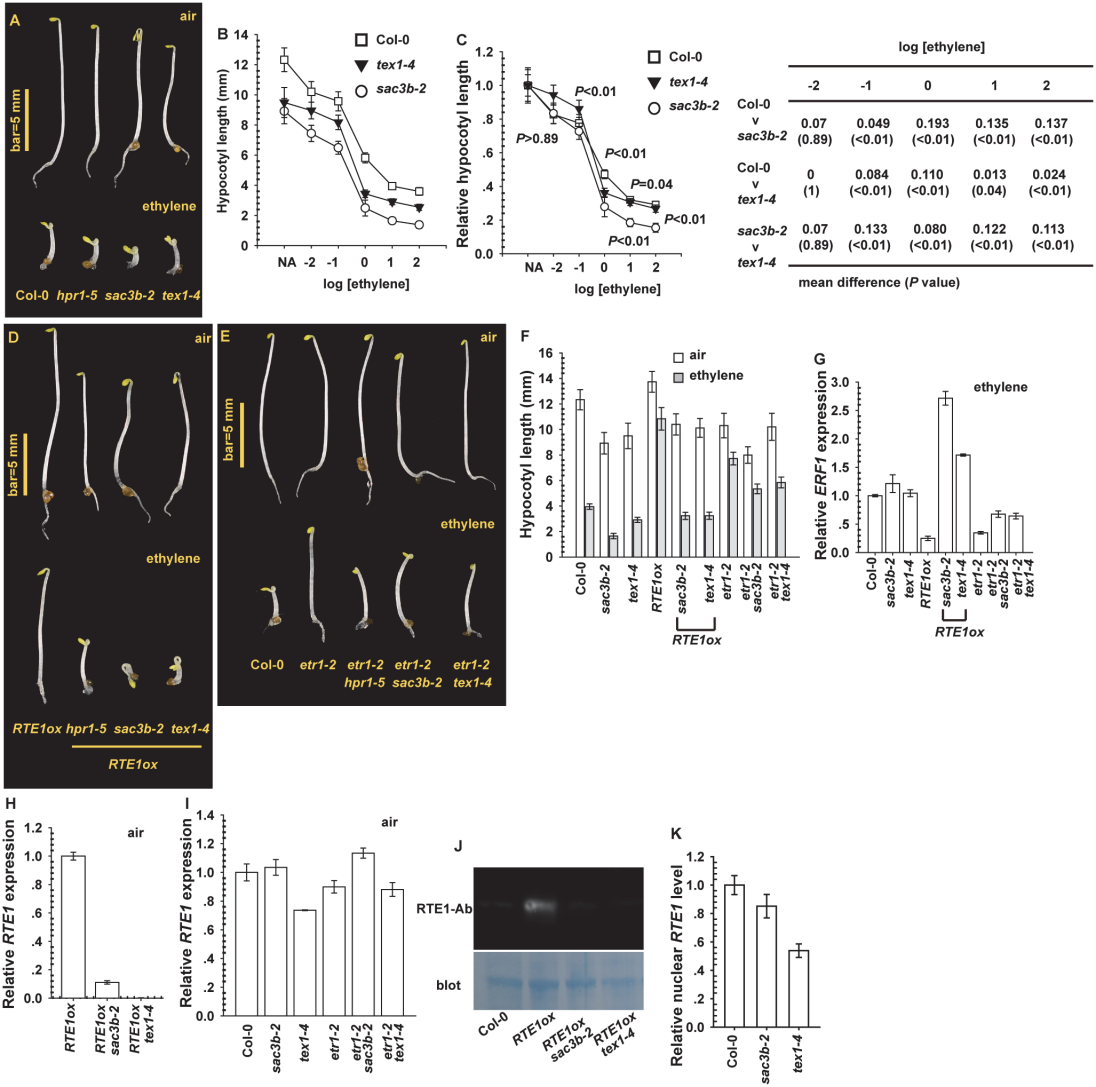
Effect of *sac3b-2* and *tex1–4* alleles on *RTE1* expression. (A) Phenotype of etiolated *sac3b-2* and *tex1–4* seedlings and wild-type (Col-0) and *hpr1–5* seedlings. Ethylene dose–response assay of hypocotyl length (B) and normalized hypocotyl length (C) for wild-type (Col-0), *sac3b-2*, and *tex1–4* seedlings. Mean difference and statistical significance were estimated by Scheffe test (α = 0.01). Ethylene concentration is presented as the logarithm of ethylene (in μL L^-1^). Phenotype of etiolated seedlings for the indicated genotypes (*sac3b-2* and *text1–4*) in *RTE1ox* (D) and the *etr1–2* allele (E), and hypocotyl length (F) and *ERF1* level (G) for the corresponding seedlings. *RTE1* level in *RTE1ox* seedlings with the s*ac3b-2* and *text1–4* alleles (H) and in *sac3b-2* and *tex1–4* seedlings with and without the *etr1–2* allele (I). (J) Immunofluorescence of RTE1 level in *RTE1ox* and the indicated genotypes with the *sac3b-2* and *tex1–4* alleles. (K) Nuclear *RTE1* level in *sac3b-2* and *tex1–4*. Blot: Commassie Blue staining to indicate the protein amount on the membrane. RTE1-Ab: a monoclonal antibody for RTE1. Data are mean±SD for seedling hypocotyls (n>30), and mean±SE for gene expression (at least 3 independent biological samples with 3 measurements for each).

We used ethylene dose–response assay to evaluate whether the loss-of-function *sac3b-2* and *tex1–4* mutations [15, 16, 24], resulting from T-DNA insertion, affect the ethylene response. Etiolated *sac3b-2* and *tex1–4* seedlings showed enhanced ethylene growth inhibition (Fig. 8A). Over a wide range of ethylene concentration, the hypocotyl was slightly shorter for etiolated mutant seedlings than the wild type (Col-0), with a shorter seedling for *sac3b-2* than *text1–4* (Fig. 8B). When normalized to the untreated seedling hypocotyl length, the relative hypocotyl length was slightly shorter for *sac3b-2* than wild-type and *tex1–4* seedlings. The *sac3b-2* and *tex1–4* mutations each conferred ethylene hypersensitivity to various degrees (Fig. 8C).

Expression of *35S:RTE1* in the wild type (Col-0) conferred ethylene insensitivity, and ethylene treatment had a very minor effect on seedling hypocotyl elongation. The transgene from *RTE1ox* (*35S:RTE1* expressed in the wild type) was introduced into *sac3b-2* and *tex1–4* by genetic crossing; homozygous *RTE1ox sac3b-2* and *RTE1ox tex1–4* were each obtained and confirmed at higher generations by genotyping. The transgene in *sac3b-2* and *tex1–4* seedlings did not confer ethylene insensitivity, and the hypocotyl was substantially shortened by ethylene treatment (Fig. 8D). The *etr1–2* ethylene-insensitive allele conferred ethylene insensitivity depending on *RTE1*, and the seedlings were moderately shortened by the *sac3b-2* and *tex1–4* mutations with ethylene treatment (Fig. 8E). The impact of the *sac3b-2* and *tex1–4* mutations was stronger on *RTE1ox* than *etr1–2*, and the seedlings were shortened to a greater degree for *RTE1ox* than *etr1–2* by each of the 2 mutations with ethylene treatment (Fig. 8F).

Consistent with the ethylene growth inhibition phenotype, with ethylene treatment, *ERF1* level was increased in wild-type (Col-0), *RTE1ox sac3b-2* and *RTE1ox tex1–4* but not *RTE1ox* seedlings (Fig. 8G). Each of the mutations also increased *ERF1* level in *etr1–2*, to a level lower than that for the ethylene-treated wild type (Fig. 8G). Therefore, *sac3b-2* and *tex1–4* each also affected ethylene insensitivity conferred by the *etr1–2* allele. Alternatively, the 2 mutations could confer increased ethylene sensitivity by mechanisms yet to be identified. *RTE1* mRNA levels in *RTE1ox* in both *sac3b-2* and *tex1–4* were substantially reduced (Fig. 8H). The native *RTE1* level was not reduced in *etr1–2* by each of the mutations; compared to the wild type (Col-0), *RTE1* level was not altered in *sac3b-2* but was reduced in *tex1–4* (Fig. 8I). As expected, RTE1 protein was detectable in *RTE1ox* but not *RTE1ox* with the *sac3b-2* or *tex1–4* mutation (Fig. 8J).

Whether *sac3b-2* and *tex1–4* could prevent *RTE1* nucleocytoplasmic export to affect RTE1 functions was evaluated by measurement of the nuclear *RTE1* level. Nuclear *RTE1* was slightly reduced in *sac3b-2* compared with the wild type (Col-0); the nuclear *RTE1* level in *tex1–4* was reduced to a greater extent (Fig. 8K), in line with reduced *RTE1* level in the mutant (Fig. 8I). The *sac3b-2* mutation does not result in bulk nuclear mRNA accumulation [16]. Our data suggested that SAC3B and TEX1 are not involved in *RTE1* export; the reduced nuclear *RTE1* level in *tex1–4* could be due to reduced gene expression or transcript stability.

### Possible Epigenetic Effects on *RTE1ox* by T-DNA Tag

Of note, on genetic crossing, the T-DNA tag containing the *35S* promoter in a mutant could have a dominant epigenetic silencing effect on the *35S* promoter-driven transgene in a transgenic line [52]. We evaluated whether the suppression of *RTE1ox* by *sac3b-2* and *tex1–4* could result from transgene silencing.

**Figure 9:**
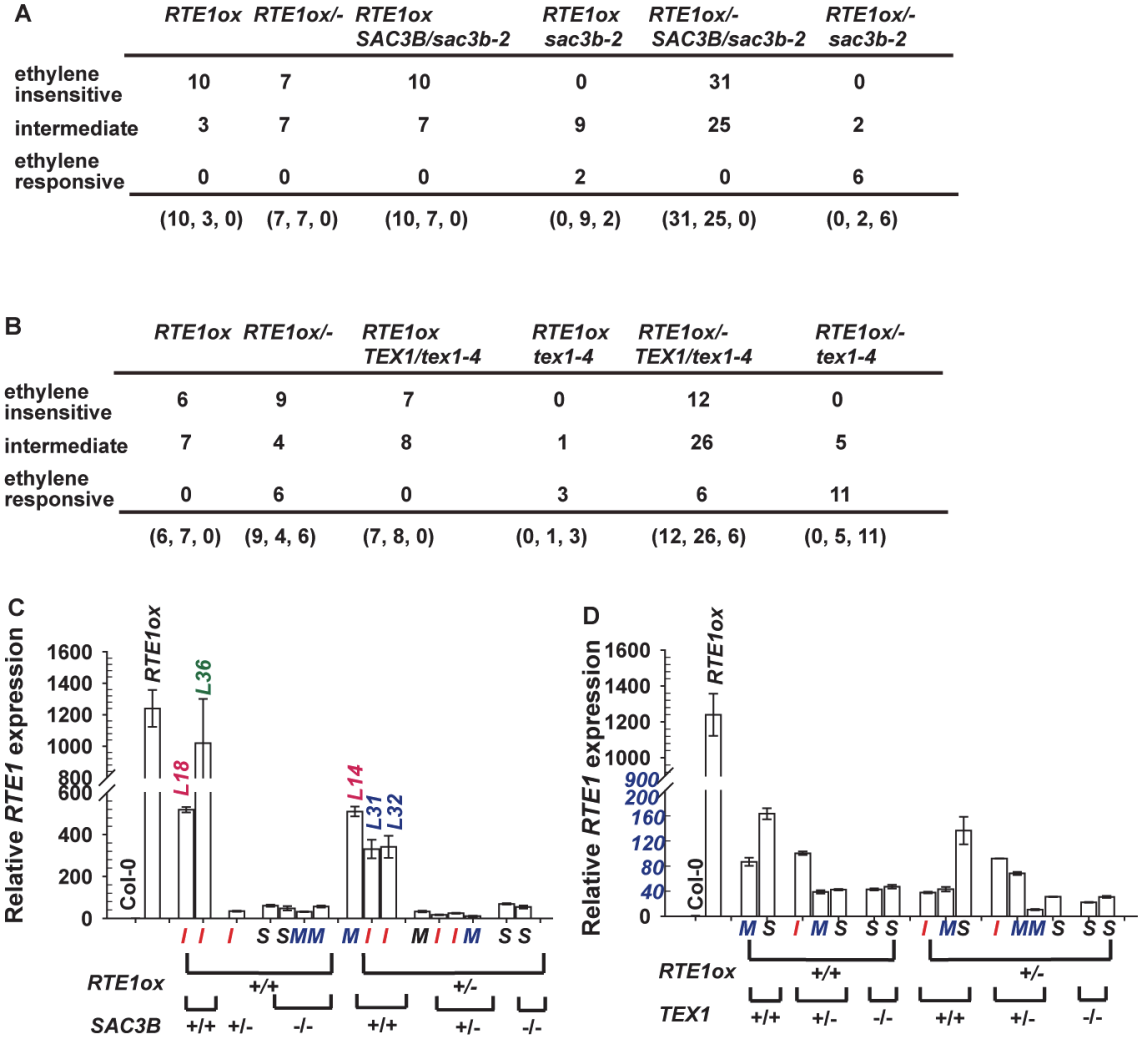
Analyses for the ethylene response phenotype and *RTE1* expression in *35S:RTE1*-containing individuals. Genotypes and ethylene response phenotypes for *35S:RTE1*-containing F2 seedlings from a genetic crossing of *RTE1ox* and *sac3b-2* (A) and *RTE1ox* and *tex1–4* (B). (C) and (D) Rosette *RTE1* levels in *35S:RTE1*-containing F2 individuals, with their genotypes and seedling ethylene-response phenotypes; *I*, *M*, and *S* indicate ethylene-insensitive, intermediate, and ethylene-responsive seedling phenotypes, respectively. *L*: line number for individual F2 lines. Data are mean±SD for 3 technical repeats, without biological duplicates.

We scored the ethylene response phenotype for the F2 seedlings of genetic crossing of *RTE1ox* with *sac3b-2* and *text1–4*. Unexpectedly, the ethylene seedling growth-inhibition phenotype was classified into 3 categories: ethylene-insensitive, ethylene-responsive, and intermediate. Individual F2 seedlings that were phenotyped were grown to the adult stage for genotyping, and we determined the association of phenotype and genotype for individuals carrying the *35S:RTE1*. The *35S:RTE1*-containing F2 generation of the *RTE1ox* and *sac3b-2* cross showed reduced ethylene insensitivity (the intermediate category), with or without the *sac3b-2* allele. In total, 2 and 9 of 11 *RTE1ox sac3b-2* individuals showed the typical ethylene-response and intermediate phenotype, respectively. For the *RTE1ox/- SAC3B/sac3b-2* heterozygote, 31 and 25 individuals showed the ethylene-insensitive and intermediate phenotypes, respectively; 6 and 2 of 8 *RTE1ox/- sac3b-2* individuals showed the ethylene-responsive and intermediate phenotypes, respectively (Fig. 9A). Ethylene insensitivity conferred by the *35S:RTE1* transgene was reduced after genetic crossing with *sac3b-2*. The *RTE1ox* suppression by *sac3b-2* observed at higher generations (Fig. 8) was not necessarily caused by the loss of *SAC3B*.

For the F2 generation of the *RTE1ox* and *tex1–4* crossing, reduced ethylene insensitivity (the intermediate phenotype) was not necessarily associated with loss of *TEX1*: 6 of the 19 F2 *RTE1ox/-* heterozygotes were ethylene-responsive. Among the 44 F2 *RTE1ox/- TEX1/tex1–4* heterozygotes examined, 12 and 6 had the ethylene-insensitive and-responsive phenotypes, respectively (Fig. 9B). The suppression of *RTE1ox* by *tex1–4* (Fig. 8) observed at higher generations after the genetic crossing was not necessarily caused by the loss of *TEX1*.

With lack of sufficient plant material for gene expression measurement and lack of biological replicates for each of the F2 seedling single lines, measurement of seedling *RTE1* expression to determine its association with phenotype and genotype of individual F2 seedlings was technically unlikely. Nevertheless, randomly selected single F2 seedlings that were phenotyped were grown to rosette stage for genotyping and *RTE1* expression measurement. Among the *35S:RTE1*-containing F2 rosettes of the *RTE1ox* and *sac3b-2* cross, *RTE1* levels were largely reduced regardless of the genotype and seedling ethylene-response phenotype, with one exception: the rosette *RTE1* level was slighted reduced in line *L36* as compared with *RTE1ox* level, and the seedlings were ethylene-insensitive (Fig. 9C). Rosette *RTE1* expression of two individual lines (*L18* and *L14*) was reduced to about 42% and that of another two lines (*L31* and *L32*) to about 26% of the *RTE1ox* level, the other lines showing *RTE1* expression 0.8% to 5% of the *RTE1ox* level (Fig. 9C). *RTE1* expression in the *35S:RTE1*-containing F2 rosettes of the *RTE1ox* and *tex1–4* cross was highly reduced, regardless of seedling phenotype and genotype (Fig. 9D).

The genetic analysis and gene expression measurement did not support the 2 mutations suppressing *35S:RTE1* expression. Silencing is inheritable over generations even if the silencing insert is removed [52, 53]. With variation in degrees of the ethylene-response phenotype and in *RTE1* expression in the F2 generation, we considered the possibility of epigenetic silencing of *35S:RTE1*. Whether the suppression was associated with the T-DNA tag or other mechanisms needs to be verified. A rapid increase in methylation of the *35S* promoter during vegetative growth leads to transgene silencing in *Nicotiana attenuata* lines with multiple T-DNA insertions [54]. The highly reduced *RTE1* levels in rosettes from seedlings with the ethylene-insensitive or intermediate phenotype might be due to rapid silencing of the *35S:RTE1* transgene during vegetative development but not the seedling stage, with the silencing mechanism remaining to be determined.

## Discussion

Previous studies suggested that ETR1 ethylene receptor signaling could be facilitated by RTE1 via the physical interaction of RTE1 and the ETR1 N-terminus [8, 10, 12, 32, 55]. Contrary to the extensive studies of ETR1, little is known about the regulation of RTE1 functions. We aimed to uncover components involved in RTE1 function for ethylene signaling regulation. We found that reduced *RTE1* expression was associated with the loss-of-function mutation of *HPR1*. Ethylene insensitivity prevented by the *hpr1–5* allele in *RTE1ox* was also associated with reduced *RTE1* mRNA and protein level at the seedling stage. The promoters for the native *RTE1* and the *35S:RTE1* transgene differ. HPR1 may have a role in normal *RTE1* transcription but not transcription initiation to produce the necessary amount of the protein to facilitate ETR1 receptor signaling. The RTE1 amount, although reduced, in rosette leaves could still be sufficient to promote ETR1/etr1–2 receptor signaling to prevent ethylene signaling, so the *hpr1–5* allele had little effect on ethylene insensitivity by the *35S:RTE1* transgene and *etr1–2* at the adult stage. The lack of suppression at the adult stage was not likely due to redundancy because of the absence of other *HPR1* homologs in the genome. Ethylene insensitivity of *ein2–50* and *ein3–1* was not suppressed by *hpr1–5*, which suggests specificity of HPR1 for RTE1 functions in ethylene signaling.

HPR1 shares a low degree of homology with yeast Hpr1 and is annotated as HPR1 of the THO tetrameric protein complex that forms the TREX complex together with other interacting proteins, including TEX1 [24, 25]. THO/TREX is involved in transcription/export of RNA species that remain largely to be identified; as expected, pleiotropy would occur on disruption of the THO/TREX complex. The growth inhibition and leaf serration phenotype as well as ethylene hypersensitivity in the *hpr1–5* and *hpr1–2* mutants could result from pleiotropy caused by aberrant gene expression and transcript export. This argument agrees with the pleiotropic phenotype observed in *emu*, which is an *HPR1* allele [25]. Expression of *ETR1*, *etr1–2*, *EBF1*, *EBF2* and *CTR1* was unaltered by *hpr1–5*; *hpr1–5* did not likely affect their expression to result in ethylene hypersensitivity. Whether the *hpr1* mutation could affect functions of these genes at other levels remains to be investigated.

Transcription/export involves various dynamic activities such as transcription elongation, 5’-capping, 3’-polyadenylation, RNA splicing, the assembly of messenger ribonuclearprotein particles (mRNPs), and mRNP nucleocytoplasmic export through the nuclear pore complex [27, 51]. Although bulk unidentified polyadenylated RNAs accumulated in the nucleus of the *hpr1–5* mutant, HPR1 involved in *RTE1* export was less likely because the total and nuclear *RTE1* amount were both reduced to a similar level in the *hpr1–5* mutant, so the allele did not result in nuclear *RTE1* accumulation. The reduced *RTE1* level could be due to defects in transcription initiation, accelerated transcript degradation, or attenuated transcription elongation. *RTE1* level in *hpr1–5* and *hpr1–5 RTE1ox* was reduced to a similar extent but not amount as compared with the wild type and *RTE1ox*, respectively. Therefore, its effect was not associated with the promoter that drove *RTE1* expression. The argument that HPR1 was involved in *RTE1* transcription initiation was not favored. The termination of RNA biosynthesis with cordycepin facilitated the study of RNA stability. *RTE1* reduction was identical between seedlings of the wild type (Col-0) and *hpr1–5* as well as *RTE1ox* and *hpr1–5 RTE1ox* with cordycepin treatment, so the *hpr1–5* allele did not accelerate *RTE1* degradation. HPR1 may be involved in *RTE1* transcription elongation.

Of note, the yeast *Hpr1* gene is required for efficient transcription elongation for the *LacZ* reporter driven by different promoters, and the transcription elongation in yeast *Δhpr1* cells is not affected for other sequences such as yeast *PHO5* [20]. Moreover, *EMU*, the same gene as *HPR1*, is required for the normal expression of *ERECTA* and several miRNAs [25]. These results support our argument that HPR1 is involved in *RTE1* transcription elongation but not initiation. Interestingly, the transcription elongation defect in yeast *Δhpr1* cells can be rescued by excess Sub2, an RNA helicase that may unwind the inhibitory secondary structure of a nascent RNA molecule; the RNA-DNA hybrid could also obstruct transcription progression [23, 56]. Efficient transcription elongation that requires HPR1 may be associated with the gene sequence or higher-order gene structure.

SAC3B is a component of the TREX-2 complex that couples transcription and mRNP export, and TEX1 is a THO-interacting protein and may have a role in the export of tasi-RNA precursors for *TAS1* and *TAS2* [15, 16, 24]. Defects in each resulted in growth inhibition and ethylene hypersensitivity in etiolated seedlings. Compared to the wild type, *sac3b-2* showed no reduction in *RTE1* levels, which were moderately reduced in *tex1–4*. The increase in ethylene sensitivity in *sac3b-2* was not associated with *RTE1* levels. Of note, nuclear *RTE1* level was reduced in *tex1–4* but not *sac3b-2*; TEX1 could be involved in nuclear *RTE1* expression or degradation but not nucleocytoplasmic export.

*RTE1* overexpression with the *35S:RTE1* transgene was substantially prevented in *sac3b-2* and *tex1–4* at higher generations after genetic crossing. Genetic analysis did not support the association of *RTE1ox* suppression with either mutation. Given that individual F2 *RTE1ox sac3b-2* and *RTE1ox tex1–4* seedling lines showed reduced ethylene insensitivity (the intermediate phenotype) as well as the typical ethylene-response phenotype and that some of the F2 *RTE1ox* individuals showed reduced ethylene insensitivity, the suppression more likely resulted from epigenetic effects on *35S:RTE1* expression. We observed strong *RTE1ox* suppression in *sac3b-2* and *tex1–4* (Fig. 8) at higher generations after genetic crossing; the epigenetic suppression could be strengthened at higher generations or the specific lines may not be representative for a population. The T-DNA tag in *sac3b-2* and *tex1–4* could confer the epigenetic silencing effect on *35S:RTE1* expression. Most of the individual F2 lines with ethylene-insensitive or intermediate phenotype at seedling stage showed highly reduced *RTE1* levels at rosette stage. The seedling *RTE1* levels were not measured because of technical limitations; nevertheless, the lack of association of seedling ethylene-response phenotype and rosette *RTE1* levels in these individual lines may be due to rapid epigenetic silencing of the *35S:RTE1* transgene during vegetative development, as observed in *N. attenuata* lines with multiple T-DNA insertions [54]. Mechanisms involved in the differential *35S:RTE1* transgene silencing in *sac3b-2* and *tex1–4* remain to be determined.

The transcription/export of RNA species involving the THO/TREX complex remains to be determined in plants and other organisms. In yeast, approximately 20% of the genome and 36% of transcription events are associated with the conserved mRNA export factors Yra1 and Mex67, which supports a combinatorial model for involvement of multiple pathways for mRNA export [57]. In *Drosophila melanogaster*, less than 20% of the transciptome is regulated by THO, as revealed by gene expression profiling in cells depleted of THO components [58]. Of the 20%, less than 12% may require THO for export. THO may not be involved in the transcription and export of most mRNAs in *Drosophila*. These studies provide an explanation for why defective HPR1 results in pleiotropy but not lethality and for why our study revealed a role of HPR1 in *RTE1* transcription elongation but not export. A small fraction of the transcriptome may depend on the THO/TREX complex in Arabidopsis.

**Figure 10:**
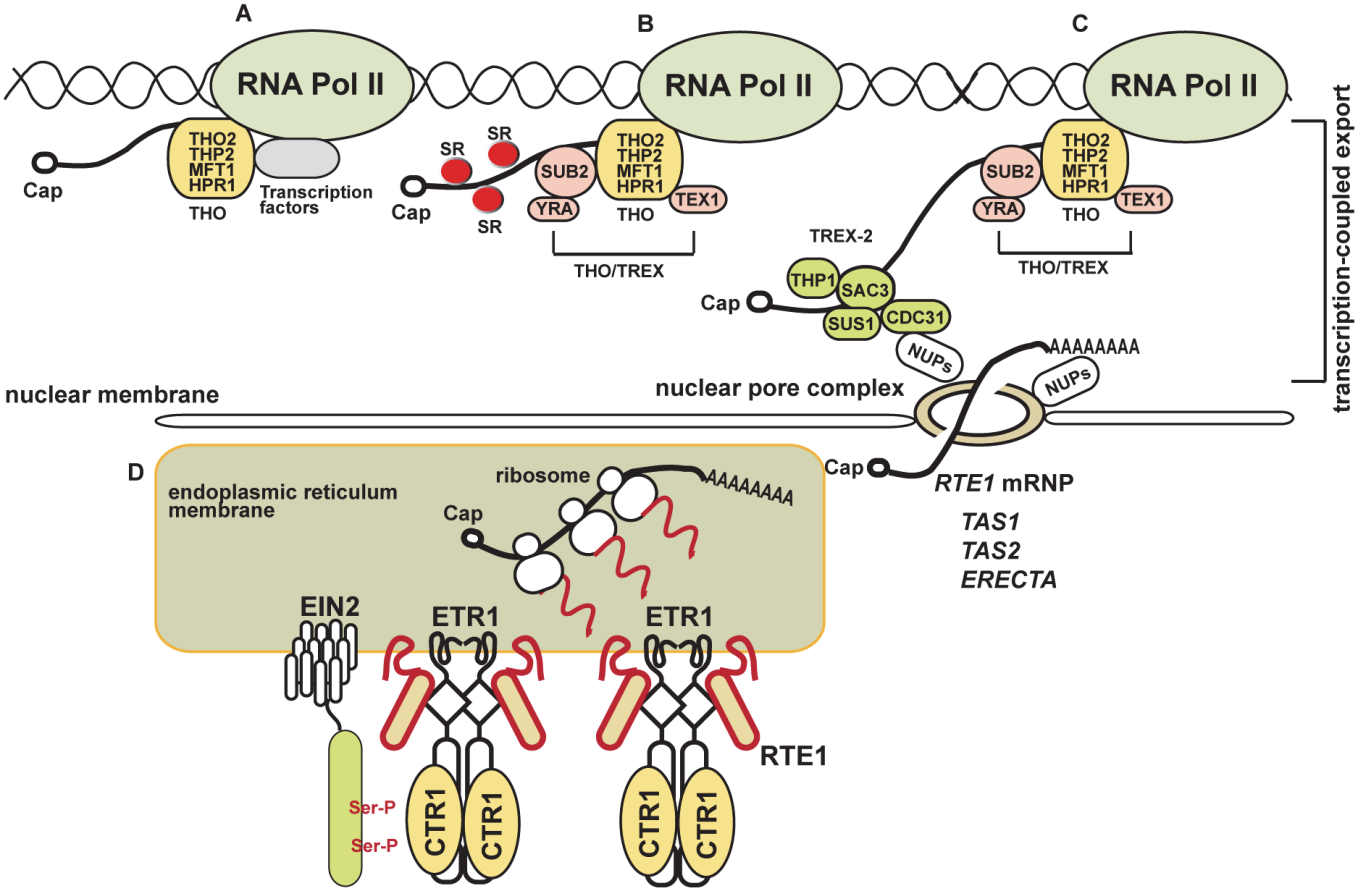
A model for the involvement of the THO/TREX complex component HPR1 in *RTE1* transcription and ethylene signaling regulation. (A) THO is associated with RNA polymerase II, possibly with transcription factors, for transcription. (B) On transcription progression, different components are recruited for mRNA transcription and processing; the SUB2-YRA dimer is recruited to form the THO/TREX complex and TEX1 is a component associated with the complex. SERINE-ARGININE–RICH proteins (SRs) are involved in RNA splicing and co-localize with the THO/TREX complex at nuclear speckle domains. (C) RNA transcription is coupled to TREX-2 (consisting of SAC3, THP1, SUS1, and CDC31) for nucleocytoplamic export through the nuclear pore complex (NPC) via the tethering of TREX-2 with nucleoporins (NUPs). Normal expression of *ERECTA* [25] and *RTE1* requires HPR1, and siRNAs derived from *TAS1* and *TAS2* involves HPR1 and TEX1 [15, 24]. *RTE1* ribonucleoprotein particle (RNP) export could be mediated by other components. (D) Once exported for translation, the produced RTE1 protein facilitates ETR1 receptor signaling to CTR1; EIN2 is retained at the endoplasmic reticulum on phosphorylation by CTR1 and ethylene signaling is prevented. HPR1 could be required for efficient *RTE1* transcription elongation through regions with higher-order RNA/DNA structures or a stable RNA–DNA hybrid that obstructs RNA polymerase II movement. Ser-P: phosphorylation of serine residues on EIN2.

The involvement of HPR1 in normal *RTE1* transcription reveals a role of transcriptional regulation in ethylene signaling (Fig. 10). The role for HPR1 in *RTE1* transcription elongation agrees with that of yeast Hpr1 in *LacZ* transcriptional elongation but not activation [20, 23] and that of Arabidopsis EMU/HPR1 in the normal expression of *ERECTA*, *TAS1*, and *TAS2* [25]. The THO/TREX component TEX1 had a role in nuclear *RTE1* level as well as *TAS1* and *TAS2* expression [15, 24]; how TEX1 is involved in *RTE1* expression remains to be investigated. Components of the THO/TREX complex have selective roles in the transcription export process of specific genes; the *hpr1–5* mutation affected bulk mRNA but not *RTE1* export, and normal *RTE1* transcription elongation required HPR1. The assembly of mRNA and RNA-binding proteins into mRNPs is dynamic; the THO/TREX complex could be involved in *RTE1* transcription but not its export. Conceivably, the nucleocytoplasmic export of *RTE1* could be mediated by other pathways. Defect of the RNA-export TREX-2 complex component SAC3B had little effect on nuclear *RTE1* level; *RTE1* export may not require SAC3B, in agreement with the lack of bulk mRNA accumulation in *sac3b-2* [16].

Except for the possible role of HPR1 in *RTE1* transcription, with a bulk mRNA accumulation in the nucleus of *hpr1–5*, the suppression of *RTE1* by *hpr1–5* could be due to nuclear accumulation of the affected transcripts that are required for RTE1 functions. Alternatively, normal transcription of genes involved in RTE1 functions could be affected by the *hpr1–5* mutation, for an indirect effect on RTE1 functions. One of the possible candidates could be cytochrome *b*_5_ (Cb5), which interacts with RTE1 to facilitate ETR1 receptor signaling at the ER membrane [59]; if Cb5 is affected by *hpr1–5*, RTE1 functions would be weakened.

## Materials and Methods

### Plant Materials and Phenotyping

Arabidopsis seeds were stratified at 4°C for 3 days (72 hr) before germination at 22°C for 80 hr in the dark (for phenotyping etiolated seedlings) or 7 days at 25°C with illumination (for phenotyping light-grown seedlings). Adult plants were grown at 25°C with fluorescent light (16-hr light/ 8-hr dark) and phenotyped 4 weeks after germination. The ethylene concentration for treatment was 10 μL L^-1^, unless otherwise specified. For the ethylene dose–response assay, stratified seeds were germinated in the dark with the necessary amount of ethylene for 80 hr, then seedling hypocotyls were measured as described [33, 55] by use of Video tesT (Moscow). For the senescence test, 4-week-old adult plants were placed in an air-tight container with ethylene (100 μL L^-1^) for 4 days, and chlorophyll content was measured as described [60]. For cordecypin treatment [46], Arabidopsis seedlings were pre-incubated for 30 min in incubation buffer, and aqueous cordecypin solution was added to a final concentration of 0.6 mM for incubation for the necessary time. *hpr1–5 35S:GFP-RTE1* was obtained by crossing *hpr1–5* and a wild type (Col-0) carrying the *35S:GFP-RTE1* transgene; thus, the transgene was expressed at the same locus.

### Mutagenesis

About 20,000 *RTE1ox* seeds were treated with ethyl methanesulfonate (EMS; 0.1% w/v) in the dark for 14 hr at room temperature, then washed with continuous flowing water for 2 to 4 hr. The treated seeds were grown, and M2 seeds were harvested for genetic screening.

### Nuclei Isolation

Nuclei were enriched and purified as described [61]. In brief, 1 g Arabidopsis tissues was ground into fine powder in liquid nitrogen, homogenized with 3-ml extraction buffer, and filtrated through miracloth (Calbiochem). An amount of 10% Triton X-100 was gradually added into the resulting filtrate to a final concentration of 1%, and the filtrate was incubated on ice for 15 min. An amount of 30% and 80% percoll was prepared with the gradient buffer, with 250 μL 30% percoll at the bottom of a 2-ml centrifuge tube, then 80% percoll was added under the 30% percoll. An amount of 1.4-ml infiltrate was added to the top of the percoll layers. The tube was centrifuged at 1,000×g for 30 min at 4°C, and nuclei were enriched at the interface of the 2 percoll solutions. The nuclei were collected carefully and added with gradient buffer to a volume of 500 μL. An amount of 250 μL 30% percoll was added to the bottom of a tube and the solution was centrifuged at 1,000×g for 10 min at 4°C. After centrifugation, the nuclei were collected as the pellet and resuspended with 200 μL nuclei storage buffer.

### Gene Expression Measurement

Gene expression was measured by quantitative RT-PCR (qRT-PCR) with StepOne Plus (Applied Biosystems) and TaKaRa SYBR Premix Ex Taq. To measure ethylene-induced *ERF1* expression, plants were treated with ethylene (10 μL L^-1^) for 4 hr. The primer sequences were for *ERF1*, F (5’-TTTCTCGATGAGAGGGTC-3’) and R (5’-AAGCTCCTCAAGGTACTG-3’); *RTE1*, F (5’-GATAGAACCAAGTGTTGC-3’) and R (5’-GCAACAAACGAATGGCAG-3’); *GFP-RTE1c-RT*, F (5’-ACAGCTGCTGGGATTACACAT-3’) and R (5’-ACACCACCCTGTCTTCAACAT-3’); *ETR1*, F (5’-GCCATCTCCAAGAGGTTTGTGAA-3’) and R (5’-CCGTTCTCATCCATGACAAGA-3’); *CTR1*, F (5’-GGCTTATGATGTGGCTAAGG-3’) and R (5’-GCAGCTACAACCTGAGC); *EBF1*, F (5’-GGAGATTGATGTTCCTTCCAAGA-3’) and R (5’-CAATAGACCGAAGACCAAGATC-3’); *EBF2*, F (5’- CTTCAGATTTAGTGGTGATGAAG-3’) and R (5’- CAAGCACTCCTCTCTTGTCCA-3’), and *tubulin* (*TUB*), F (5’-TCAAGAGGTTCTCAGCAGTA-3’) and R (5’-TCACCTTCTTCATCCGCAGTT-3’) (the calibrator). The calibrator for gene expression by cordycepin treatment was *eIF4A* and the primers were F (5’-TGACCACACAGTCTCTGCAA-3’) and R (5’-ACCAGGGAGACTTGTTGGAC-3’). For measuring *UBIQUITIN* and *TUBULIN* transcript copy number, cDNAs of known amount for each gene were serially diluted as the template, and a standard curve (R^2^>0.99) was drawn for qRT-PCR. *UBIQUITIN* and *TUBULIN* cDNAs reverse-transcribed from 500 ng RNA with use of oligo(dT) were estimated for copy number according to the standard curves (R^2^>0.99). The primer sequences for *UBIQUITIN* were UBI-F (5’-ATGGAAAATCCCACCTACTAAATT-3’) and UBI-R (5’-TTGAACAACTCGTAGCAACTCATC-3’).

### Transformation and Transgenes

Transformation by *Agrobacterium* was performed as described [62], and the resulting homozygous transformation lines were characterized with T3 and higher generations. For transient expression, tobacco or Arabidopsis leaves were infiltrated with *Agrobacterium* containing the necessary clones; expression of the fluorescent proteins with the transgenes was observed 2 to 3 days after infiltration. To clone the *HPR1* promoter, the primers for PCR were HPR1p-F-EcoRI (5’-CGGAATTCAGTTTTTCTTCAAGTGACGAGAA-3’) and HPR1p-R-HindIII (5’-AGAAGCTTTAATTTAGGGTGGATCCGGAT-3’). The genomic *HPR1* fragment (*gHPR1*) was cloned by PCR with the primers gHPR1-F-HindIII (5’-AGAAGCTTAAATTGTTCTTCCTCCACTCTT-3’) and gHPR1-R-HindIII (5’-AGAAGCTTTCATGAGACGGGCATAGGAGGAT-3’). The *HPR1* promoter was cloned to the vector pBJ36, and the genomic *HPR1* clone *gHPR1* was cloned to the *Hind*III site of the promoter clone. *HPR1p:gHPR1* was released by *Not* I and cloned to the binary vector pMLBART. *HPR1* cDNA was cloned with the primers HPR1-c-F-BglII (5’-ATAGATCTATGGATGCATTTAGAGATGCTAT-3’) and HPR1-c-R-BglII (5’-ATAGATCTTCATGAGACGGGCATAGGAGGAT-3’), and sequence analysis identified the *HPR1a* and *HPR1b* cDNAs; each was cloned to the pRTL2 vector containing *35S:mGFP*. The resulting clones were each restricted with *Hind*III to release *35S:mGFP-HPR1a* and *35S:mGFP-HPR1b*, and the fragments were each cloned to the binary vector pCGN1547.

### Laser Scanning Confocal Microscopy (LSCM)

LSCM for the co-localization study of SR33-YFP and HPR1-mCherry involved use of an Olympus FV1000 microscope at our institution’s facility. The fluorescence produced by GFP-HPR1a and GFP-HPR1b was acquired with a DeltaVision Personal DV system (Applied Precision) as described [63]. The image was subjected to constrained iterative deconvolution by use of softWoRx (Applied Precision).

### *In Vivo* mRNA Hybridization [19]

Light-grown Arabidopsis seedlings 7 days old were fixed for 30 min (fixation buffer:heptane = 1:1) and washed with methanol twice (5 min for each wash) and with absolute ethanol 3 times (5 min for each wash). After the wash, the seedlings were incubated in ethanol:xylene (1:1, v:v) for 30 min, washed with absolute ethanol twice (5 min for each), methanol twice (5 min for each), methanol:fixation solution (1:1; without formaldehyde) for 5 min, and incubated in fixation solution for 30 min. Then seedlings were washed with fixation solution (without formaldehyde) twice (5 min each) and with Perfect Hyb Plus hybridization buffer (Sigma, Cat. H7033) for 5 min. With pre-treatment with the Perfect Hyb Plus hybridization buffer for 1 h, fluoresceine-labeled oligo(dT)_45_ or *RTE1* oligos (Invitrogen) was added to the hybridization to a final concentration of 10 nM. Two *RTE1* oligonucleotide sequences were used for hybridization, 5’-FITC-RTE1a (5’-CTCGATAGAACCAAGTGTTGCTTACCACCA-3’) and 5’-FITC-RTE1b (5’-AACATAATTACTTGATTTTTTTTTTTGA-3’), at 50°C in the dark overnight. Then seedlings were washed with 2×SSC with 0.1% SDS at room temperature, in the dark for 5 min, then twice with 0.5×SSC with 0.1% SDS at 50°C (20 min for each wash) in the dark. Hybridization was observed by LSCM.

### Immunoassay

Immunoassay and enhanced chemiluminescence (ECL) was performed as described [6, 32]. The mouse monoclonal antibody for RTE1 (RTE1-Ab) was custom-made by Abmart (Shanghai). The mouse monoclonal antibody for ACTIN (ACTIN-Ab) was from Abmart. The chemiluminance was captured by use of a cold charge-coupled device (Tanon 5500 Chemiluminescent Imaging System, Tanon, Shanghai, or VersArray System, Princeton Instruments) and analyzed by use of MetaMorph (Molecular Devices) as described [6, 32] for quantification and to normalize the signal strength.

### Statistical Analysis

Data are expressed as mean±SD or mean±SE. Student’s *t* test (α=0.01) was used to compare 2 groups and Scheffe test (α=0.01) for multiple groups. For each seedling hypocotyl measurement, at least 30 seedlings were measured (n≥30).
